# 33392 Investigating the relationship between placement instability, mental health, behavioral and justice-related outcomes among sex-trafficked youth

**DOI:** 10.1017/cts.2021.618

**Published:** 2021-03-31

**Authors:** Mekeila Cook, Meagan Rainock, Breanna Thomas

**Affiliations:** 1 Meharry Medical College; 2 Vanderbilt University; 3 Indiana University

## Abstract

ABSTRACT IMPACT: This public health work contributes to the development and implementation of best practices for working with sex trafficked youth who experience placement instability and justice involvement. OBJECTIVES/GOALS: Youth removed from their home into foster care or a group home (i.e., placement instability) are vulnerable to sex trafficking. This study examines whether placement instability predicts mental health, behavioral and justice-related outcomes among sex-trafficked girls. METHODS/STUDY POPULATION: Placement instability occurs when children are temporarily or permanently removed from their home and placed in foster care or a group home. Domestic minor sex trafficking is exploitation and abuse of children for commercial sexual purposes in exchange for money or other goods/services. We hypothesize that sex trafficked girls who experience placement instability will report more mental health challenges, substance use, abuse history and justice involvement than those without placement instability. Data came from participant files in a specialty court program from 2012-2014 (N=184). Multiple sources contribute to the information contained court files; all data extracted by the research team come solely from the court files. Descriptive, bivariate, and logistic regression analyses were performed. RESULTS/ANTICIPATED RESULTS: All participants were (cis)female, 74% were African American, 96% US citizens, with average age of 16 years. Three-quarters of participants had a documented mental health challenge, such as depression and 88% reported substance use. Eighty-one percent of participants had been in a placement, with a group average of 4.5 placements. Girls with placement instability reported more mental health challenges (p<.001), substance use (p<.001), abuse (p<.001), running away (p<.001) and bench warrants (p<.001) than girls without placement instability. Logistic regression estimated housing instability was positively associated with mental health challenge, substance use, running away, number of bench warrants, and number of citations. DISCUSSION/SIGNIFICANCE OF FINDINGS: Among girls who have been trafficked, placement instability places them at greater risk for personal and behavioral challenges, including increased justice involvement. Comprehensive trauma-informed services should be provided to the family to help mitigate issues in the home.

